# The High-Risk Model of Threat Perception Modulates Learning of Placebo and Nocebo Effects and Functional Somatic Disorders

**DOI:** 10.3390/brainsci15090955

**Published:** 2025-09-02

**Authors:** Ian Wickramasekera

**Affiliations:** Department of Psychiatry and Behavioral Sciences, Eastern Virginia Medical School, Norfolk, VA 23507, USA; iaehwj38@gmail.com

**Keywords:** threat, suggestibility, placebo, nocebo, iatrogenic injury, somatization, paradoxical temperature increase, adverse childhood events, autonomic nervous system dysregulation, biological embedding

## Abstract

Threat activation or deactivation in the brain–body is associated with **learned** nocebo or placebo **somatic effects** induced by **fake invasive** medical–surgical procedures. Some **functional somatic disorders (FSDs)** originate as **acute** nocebo **somatic effects** and can become 30–50% of **chronic somatic** presentations to primary care physicians. Patients with FSD **overutilize** medical–surgical services, despite the **lack of identified pathophysiology,** and are at risk for **morbidity** from **unintentional iatrogenic injury**. The Conditioned Response Model (**CRM**) of **learning** postulates three **innate** mechanisms, **modulated** by **trait hypnotizability**, which drive placebo and nocebo **somatic effects** and FSD. The High Risk Model of Threat Perception **(HRMTP)** postulates **10 psychosocial risk factors** that ***modulate threat perception***, driving *placebo and nocebo* **somatic *effects*** *and* **biologically embedded FSD**. **Psychosocial factors** and the **trait** of **high and low hypnotizability modulate threat** and are postulated to **reduce** ***heart rate variability*** **(*HRV*)**, ***inducing autonomic nervous system*** **(*ANS*)** ***dysregulation***. ***Reduced HRV was found in*** a large (N = 6,891) sample of patients with **FSD**. A total of 50% of patients with **FSD** with chronic pain (n = 224) **without identified pathophysiology** had a **Paradoxical Increase in hand Temperature (PTI**) during experimental **threat** induction. The HRMTP predicts that PTI associated with **ANS dysregulation** is associated with the **risk factor Adverse Childhood Experiences (ACEs)**. This **ACE** prediction was **independently** confirmed. Learning predicts that **threat** activation by **unconscious neutral stimuli (CS)** can **amplify** nocebo and FSD and can **negate** placebo effects in clinician–patient relationships. Identifying **psychosocial risk factors** that **modulate threat perception** enables the diagnosis of FSD by **inclusion** and not simply by **excluding pathophysiology**.

## 1. Introduction

The analysis of life and survival, even down to the level of the immune system, is about “identifying and neutralizing **threats** from other species” [1]. Immune mechanisms operate behaviorally through threat avoidance by associative Pavlovian learning [2,3]. **Threat (HPAA)** learning appears to be mediated by **unconscious automatic** amygdala brain circuits, but fear learning is apparently mediated by neocortical circuits [4]. Acute **threat** in humans [5,6,7,8] is apparently mediated **automatically and unconsciously** by the sympathetic division of the autonomic nervous system (ANS) through cortisol. Associative **emotional** learning of **threat activation or deactivation** in the **brain and body**, manifested as **clinical** nocebo and placebo **somatic effects**, are postulated to be associated with the modulation of the hormones of the **neuroendocrine system**, specifically **cortisol and the immune system** [6,7,9,10,11,12,13]. **High and low cortisol secretion** implicates the **hypothalamic–pituitary–adrenal axis (HPAA**) and **pro- and anti-inflammatory cytokines** [6,9,14,15,16,17].

This article is primarily a selective review of 1) Placebo, 2) Nocebo and 3) Functional Somatic Disorders (FSD) focusing primarily on **two theoretical models** (Conditioned Response Model of Learning-CRM and the High Risk Model of Threat Perception-HRMTP) which integrate **the acquisition, extinction and modulation** by 10 psychosocial risk factors, of the above 3 clinical phenomena in terms of **autonomically** (ANS) mediated human **emotional learning**.

Reduced heart rate variability (HRV) or **low parasympathetic activity** is an independent risk factor associated with a 2-year risk of sudden death [18,19,20]. Reduced HRV is also associated with functional somatic disorders (FSDs) in Denmark, with a large sample (n = 6891) of patients [21]. The High Risk Model of Threat Perception—HRMTP [22,23,24,25,26,27,28,29,30,31,32,33,34] postulates that trait high and low hypnotizability, but not moderate hypnotizability (68% of the general population), is a risk factor for (1) sympathetic hyperreactivity and delayed parasympathetic recovery and eventually for (2) dysregulation of the autonomic nervous system and PTI [35,36,37]. Jorgensen & Zachariae [23] using normal healthy college students tested the above hypotheses with HRV and EDR (electrodermal) data and experimentally induced cognitive and emotional threat and found that there was an apparent potential for sympathetic hyperreactivity and delayed parasympathetic recovery and a potential for ANS dysregulation in trait high and low, but not moderate, hypnotizability. Jorgensen and Zachariae’s [23] data (Figure 3 and Figure 4) appeared to confirm the HRMTP hypothesis of **reduced HRV in trait high and low hypnotizability** but not moderate trait hypnotizability. Jorgensen & Zachariae [23] also appeared to confirm the hypothesis of a distinct *quadratic relationship* between trait hypnotizability and threat perception, postulated by the HRMTP [24,27,30,31,32,33].

Greenleaf et al. [22] also found a **quadratic relationship** between **trait hypnotizability** and **threat** of surgical incision in coronary bypass surgery patients, modulating their **blood pressure in the ICU**, and reported their outcome data in terms of **blood pressure medication utilization** and speed of **recovery** and **discharge** from the **ICU** and hospital. Perlstrom and Wickramasekera [38] also found a **quadratic relationship** between **trait hypnotizability and EEG**-**defined primary insomnia** and **threat perception** (High Neuroticism), **without identified pathophysiology**. Younger et al. [34], also using healthy college students and **psychometric–behavioral** measures of **somatic symptoms**, confirmed a **dose**–**response or linear relationship** between **trait hypnotizability** and **somatic symptoms** but not the predicted quadratic model of **ANS hyperreactivity** and eventual **ANS dysregulation** [29,30,31,33,35,36]. The Jorgensen & Zachariae [23] study and Greenleaf et al. [22] used the predicted **ANS measures of threat perception** [24,27,33], whereas the Younger et al. [34] study used only **behavioral measures** of threat perception. Two **large major meta-analyses** of the **efficacy of trait hypnotizability–suggestibility** in **reducing threat** in **experimental** [39] **pain (n = 3632) and clinical** [40] **pain** (in 42 studies) found **a linear relationship between these two predicted variables**. **A linear relationship** between **trait hypnotizability** and **threat** perception in **somatic symptom modulation** was found by multiple other **clinical** and meta-analytic studies [41]. For example, the following studies found **a linear relationship** between **trait hypnotizability–suggestibility** and clinical or experimental **threat** perception in the following somatic symptoms: **anticipatory nausea and vomiting** [42,43], **chronic tension headache** [44], **Conversion Disorder** [45], **moderate obesity** [46], **surgical candidates with morbid obesity** [47], **somatization** [48], **chronic pain** [49], **primary care somatization patients** [50] **and acute stress disorder** [51].

George Engel [52], in a salient paper in ***Science***, proposed a *new medical model*, but he did **not specify** or **operationalize** the **psychosocial factors of his model**. Nor did he specify the **mechanisms of learning** [53,54] through which **threat perception** and *activation* of the **HPAA** could be **modulated** by **psychosocial risk factors** to induce **stress**-**related somatic symptoms (SRSSs)**, **without identified pathophysiology** [24,25,26,27,28,29,30,31,32,33] or, as labeled today, **functional somatic disorders—FSDs** [55]. **Threat perception (HPAA)** pierces the skin, **invades** the **brain and body** and is correlated with **somatic** symptoms [6,7,56,57] which are postulated to be **modulated** by **ten semi-orthogonal empirically established psychosocial risk factors** specified by the **High Risk Model of Threat Perception (HRMTP)** [24,25,26,27,28,29,30,31,32,33]. HPAA **activation is correlatively** linked to chronic **SRSS** or somatization [24,30,33,58,59]. Activation or deactivation of **threat** (HPAA) perception by **fake invasive medical–surgical procedures** [5,60] drive **learned** *placebo and nocebo* **somatic effects** and may contribute to ***chronic*** *functional somatic disorders (FSDs*) **without** identified pathophysiology [30,32,33]. Placebo and nocebo somatic effects and functional somatic disorders are postulated to be modulated by ***learned* threat perception**, which is modulated by the **Predisposing**, **Triggering and Buffering** psychosocial risk factors of the **HRMTP** [27,30,32,33]. **Threat** perception is a link through the associative (CS-US) and other learning of placebo and nocebo **somatic effects** and functional somatic disorders [33,53,54,61,62] from *mental to physical* health [24,30,31,35,36,55].

The primary Predisposing risk factor, trait hypnotizability–suggestibility, of the HRMTP is postulated to modulate invasive threat perception (HPAA), somatic symptoms and experimental and clinical pain perception [22,23,24,33,34,35,36,37,40,63,64,65,66,67]. Acute and chronic threat perception is postulated to be associated with both *hyper*- and *hypo*activation of the HPAA and this variability is modulated by several factors, like the type of stressor, the type of personality and the timing of the stressor [68]. Threat and acute pain activates the hypothalamic–pituitary–adrenal axis (HPAA) to secrete cortisol, and variability in *chronic* cortisol secretion is predicted by the HRMTP to contribute to dysregulation of the autonomic nervous system and paradoxical temperature increase—PTI—in chronic pain [6,7,17,35,36,37] and can induce other stress-related somatic symptoms (SRSSs) [17,24,27,30,31,33,36,37] or FSDs [55].

**Reduced heart rate variability (HRV)**, consistent with sympathetic (SNS) ***predominance***, was found in a large-scale study in Denmark of people with **FSD** (N = 6891) and also was found in several previous meta-analyses of HRV and FSD [21]. This **recent *sympathetic*** ***predominance*** finding in a large FSD sample is consistent with the HRMTP’s prediction of **sympathetic hyperreactivity and delayed parasympathetic recovery** in the primary Predisposing risk factor **trait high hypnotizability in FSD or SRSS** [22,23,31,33,35,36,64].

**Trait low hypnotizability** appears related to **incongruence in measures of ANS reactivity and emotional hypoactivation** of the HPAA [23] on **conscious verbal report measures** but **not on direct ANS measures** of **threat** perception [27,69,70,71]. Trait **low hypnotizability** is apparently related to **trait High Alexithymia**, **defined as “without words for feelings”** [72,73]. High Alexithymia is **negatively** correlated with trait **high hypnotizability** [72,74,75]. Threat perception in FSD appears associated with **incongruent emotional anomalies** in vagally modulated gut disorders specified by Porges [76] like IBS and specifically with **affective agnosia** [72,75,76,77,78,79].

Hence, the HRMTP predicts that chronic *hyper- or hypoactivation* of threat (HPAA) and emotional anomalies like affective agnosia [78], as clinically observed in many chronic pain patients with trait high or low hypnotizability–suggestibility and FSD, will eventually induce dysregulation of the ANS [30,35,36,37,79,80,81]. Normal organ function, termed homeostasis, is apparently disrupted during allostasis or adaptation to chronic threat through change [6,7]. Allostasis requires reciprocal interactions between the ANS and the immune systems [7,82]. *Chronic threat perception* is apparently associated with dysregulation of the ANS and PTI during adaptation to chronic threat, termed allostasis [7,82].

## 2. Is Paradoxical Skin Temperature Increase (PTI) During Threat Perception in Chronic Pain Linked to Dysregulation of the ANS?

We now review the first empirical confirmation [37] of HRMTP’s prediction of dysregulation of the autonomic nervous system (ANS), during experimental threat induction in patients (N = 224) with FSD with chronic pain (persistent for >6 months without positive response to conventional medical–surgical therapy) and without identified pathophysiology [30,37]. In an electrically shielded and temperature-controlled psychophysiology lab, an experimentally induced emotional–cognitive *threat state*, measured with the electrodermal response—SCL—a correlate of state negative affect [83,84,85,86], was induced by a simple timed mental arithmetic stress test. A subset of this group of 224 FSD adult chronic pain patients (mean age = 38.05 years, SD = 11.92 years, men = 83 and women = 141) responded to *emotional–cognitive threat* with a *paradoxical left*-hand temperature *increase* (PTI) during threat induction. This PTI was observed in 49.4% of male patients and 42.6% of female patients during the standardized experimental *emotional* threat induction procedure, *validated* by their increased Skin Conductance Level (SCL) during their increased left-hand temperature response. Concurrently, we also monitored several other parameters of peripheral physiological function (*BVP*, *HR*, *frontalis EMG*, etc.) that responded *normally*. This *paradoxical left-hand temperature ANS* response to threat (HPAA) *induction* appears restricted to the peripheral vascular hand temperature of these chronic FSD pain patients. A validity check of their SCL, which is primarily sympathetically innervated [84,87], and other physiological measures found normal SNS activation during (1) baseline, (2) threat induction (HPAA) and (3) return to baseline. The PTI was also *unrelated* to *regression to the mean* as indicated by data analysis and the Blomqvist [88] statistical test. The PTI is postulated to be a specific peripheral vascular response and an apparent objective marker of a predicted *dysregulated ANS* response to *chronic threat perception* [31,36,37] related to an apparent anomalous peripheral *increase in vagal tone* during threat perception.

The PTI response to **threat** perception may be an ***automatic*** ***unconscious*** **protective**–**dissociative response** [89] *mediated partly* by the interaction of **trait** (1) ***hypnotizability–suggestibility*** ***and*** (2) ***trait High Neuroticism*** [30,90] of the HRMTP. In the medical literature, this anomalous phenomena of PTI has been previously observed and is called “**idiopathic flushing**” [91,92,93] and PTI has been observed even after ***sympathetic*** ***blockade*** [92] and also during “panic attacks.” The HRMTP predicts and has found the PTI response in patients with chronic threat-related somatic symptoms (SRSSs) like **chronic pain**, **IBS and morbid obesity** in candidates for GI surgery [28,37,47,90]. The HRMTP predicts that **PTI will be observed in FSD** [55] and specifically those FSDs with the Triggering risk factor **Adverse Childhood Experiences (ACEs)** or other “**biologically embedded body memories**” [6,57] of adversity [94] or **trauma blocked from consciousness** [28,29,31,36,37,90,95].

An independent PhD dissertation [96], using our experimental threat induction procedure [37] with experimental (n = 20) and control (n = 20) groups, **replicated the PTI observation**. The study found that PTI occurred significantly (*p* = 0.0266) more frequently in the **Adverse Childhood Experiences risk factor group** (**abused sample**) than in the non-abused sample. This PTI response can be **inexpensively** tested in a psychophysiology lab and **confirmed or falsified** with an **FSD sample** of **chronic pain patients** with a history of **Adverse Childhood Experiences**.

## 3. The HRMTP Modulates Functional Somatic Disorders and Placebo and Nocebo Somatic Effects

“*Sometimes it is more important to know what kind of patient has a disease than what kind of disease the patient has*.”

—Sir William Osler

The HRMTP seeks to operationalize Osler’s intuition that sometimes the interaction of the **psychosocial features of a patient**, with the **threat** (HPAA) activating **invasive** clinical investigation (US-UR) of the patient in a medical–surgical context [53,54,61], may account for more variance in the measurement of clinical outcome than the **identified pathophysiology of their disease**. In the biomedical model, illness and somatic symptoms are driven primarily by **identified pathophysiology**. The HRMTP postulates that the penetration of the skin, brain and body *by* **threat perception** (HPAA), in the **increasingly invasive medical–surgical context**, is modulated by 10 **semi-orthogonal** empirically established psychosocial risk factors (**modulating human emotionality**) that are postulated to modulate **ANS reactivity**. These 10 psychosocial risk factors fall into three categories, **Predisposing**, **Triggering and Buffering risk factors,** and these risk factors can **interact with chronicity** in the same patient to generate ***morbidity*** ***and mortality***.

The HRMTP’s **Buffering or protective** psychosocial factors include the following: 1. **high social support** (S.S.), 2. **High Approach** and Low Avoidance Coping Skills (CS) and 3. **high trait positive affectivity** (P.A.). If **perceived** social support is low, there is an increased risk of patient *progression* of **morbidity** or **mortality** even in *stress-related organic diseases,* like cancer and cardiovascular disease [97,98,99]. Smith et al. [100] have proposed multiple mechanistic causal pathways between psychosocial variables (e.g., social support) and biological variables.

**Figure 1:**
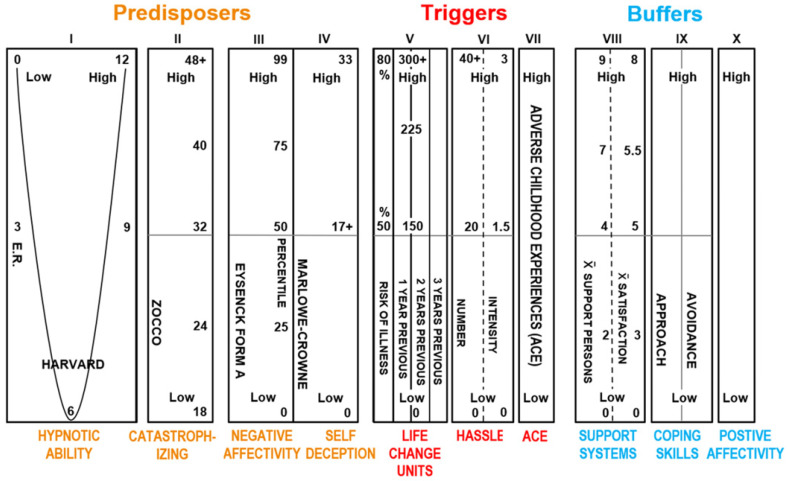
Profile of the **HRMTP**. The Predisposes can be *amplified* by the Triggers, and the Buffers theoretically *attenuate* the Predisposes and Triggers [23,24,31,33,34,36,38,44,45,47,48,49,50,64,69,70].

These 10 psychosocial risk factors are postulated to modulate threat perception in placebo and nocebo somatic effects and FSD in patients. The primary Predisposing risk factor trait hypnotizability–suggestibility can interact with threat perception in a top-down and bottom-up manner to generate somatic placebo effects [101,102,103,104,105,106], somatic nocebo effects [106,107,108,109] and functional somatic disorders [23,30,34,38,41,44,45] in patients and healthy people (Figure 1).

*A. Predisposing Risk Factors Modulating Threat and Pain Perception*

1. ***Trait hypnotizability–suggestibility*** [65,66,67,71,110], related trait **Absorption** [111] and trait **Alexithymia** [27,72,75,78] are **three closely related personality trait factors** that are postulated by the HRMTP to be psychosocial personality mechanisms modulating (1) ANS reactivity during threat (HPAA) and pain perception, (2) the generation of somatic placebo and nocebo effects and (3) FSD [55]. Specifically, it is postulated [24,26,27,33] that these three related personality traits, (1) hypnotizability–suggestibility, (2) Absorption and (3) Alexithymia, **despite varied labels**, all empirically *converge* to modulate human **threat perception** (HPAA) and **pain perception** and to modulate FSD [55].
2. ***High learned catastrophizing*** [24,28,30,112,113,114,115,116,117] is explicit or implicit verbal responses to threat that reliably amplifies threat and pain perception and is measured by verbal report psychometric scales [27,114] and also apparently manifests in EEG effective connectivity measures [113]. Amygdala functional connectivity in fMRI data mediates the association between catastrophizing and threat-safety learning in chronic pain in youth [16].
3. ***High trait Neuroticism or Negative Affectivity (NA)*** [24,30,36,38,86,112,118,119] is a predisposition to amplified threat perception, independent of objective negative events. Trait or state NA can be measured with psychometric measures [86] or by the electrodermal response—EDR [36,38,83], and state NA appears associated with the default mode network (DMN) in chronic low back pain [85]. Nocebo effects are linked to high NA as a risk factor [116]. High NA is robustly related experimentally, even to the common cold ([120], NEJM) and medical illness with identified pathophysiology [121].
4. ***High trait Self-Deception (SD)*** is a trait tendency to interpret negative events or threats positively [30,33,112]. SD is measured with a High Marlowe Crowne score [122,123] or another psychometric measure of social desirability. High SD is a >17 score on the Marlowe Crowne psychometric scale and is a measure of *repressed* threat perception unrelated to the complex Weinberger hypothesis [122,124].

*B. Triggering Risk Factors Modulating Threat or Pain Perception*

1. ***Life Change Units or stressful life events (SLEs)*** have been empirically linked to the experimentally induced common cold ([120], NEJM) and to morbidity and mortality [30,33,94,125,126,127,128] and are measured by several psychometric scales [126].
2. *The density and intensity of Daily Hassles*: Accumulation of Daily Hassles inducing *threat in some people activates the hypothalamic–pituitary–adrenal axis and* can be associated with mortality and morbidity [24,30,33,129]. Hassles are measured with several psychometric scales of known reliability and validity.
3. *High adverse childhood experiences (ACEs)* are *biologically embedded* and strongly linked in adulthood to depression, smoking, substance abuse, severe obesity, heart disease, cancer, chronic lung disease and reduced life span [6,7,33,57,130,131,132] and are measured by several psychometric scales.

*C. Buffering Risk Factors Modulating Threat or Pain Perception*

1. ***High*** ***social support*** [24,26,30,33,99,100,133] is measured by several verbal report psychometric scales and is reliably correlated with many physical diseases, including cardiovascular and cancer diseases, in replicated studies of morbidity and mortality mediated by threat (HPAA) and other neuroendocrine measures.
2. ***Approach*** ***and Avoidance Coping Skills*** [133,134,135] are measured by Approach and Avoidance psychometric scales [136] and are related to amplified Approach Coping Skills in positive mental and physical health outcomes [133]. Two-factor learning theory [27] predicts that avoidance behavior [137] is associated with many mental disorders and avoidance coping is postulated to be associated with FSD, specifically chronic pain [27,32,138]. Approach coping and placebo effects are associated with “safety cues” and fear extinction [27,139] and reduced somatization and reduced FSD.
3. ***High*** ***positive affectivity*** [30,140,141,142] is measured with several psychometric verbal report scales and is reliably related to positive mental and physical clinical health outcomes [140]. High positive affectivity appears to buffer nocebo effects [141]. Two studies [141,142] recently confirmed the HRMTP’s prediction that experimentally induced trait or state positive affectivity would buffer or reduce nocebo effects.

The replicated empirical support for *each* of the above 10 postulated risk factors is *too extensive* to review here and is reviewed elsewhere [6,23,24,26,27,28,30,32,33,35,36,57,72,86,123,133,140,143]. The review of the supporting evidence for the HRMTP is restricted to trait hypnotizability–suggestibility, the primary Predisposing risk factor and its two related traits, Absorption and Alexithymia.

## 4. Primary Predisposing Risk Factor Trait Hypnotizability–Suggestibility Modulates Threat Perception (HPAA) and the Expression of Other Predisposing, Triggering and Buffering Risk Factors

The psychophysiological ***expression*** of the Triggering risk factors, adverse childhood experiences (ACEs) and adult stressful life events (SLEs), and the Buffering risk factors (e.g., social support, trait high positive affectivity, etc.) are predicted by the HRMTP to be **expressed and modulated** by the primary Predisposing risk factor, **trait hypnotizability–suggestibility** [30,65,110]. ***Trait*** ***high and low*** **hypnotizability–suggestibility**, **but not moderate trait hypnotizability** (approximately 70% of the general population), appears to **quadratically** modulate **ANS sympathetic threat (HPAA) reactivity** (e.g., blood pressure lability, EDR and HRV) during **invasive** cardiac bypass surgical incision in patients [22] and to experimentally induced **distinct patterns** of **ANS reactivity**, measured by heart rate variability (HRV) and electrodermal skin conductance (EDR), **to *emotional*–cognitive threat** induction in normal college students [23,64]. It is postulated that personality trait (a) **high hypnotizability–suggestibility** is associated with ANS ***Hype****r-responsiveness* and (b) **trait low hypnotizability** with **emotional–cognitive incongruencies** [26,27,69,70] and ANS ***Hypo****-responsiveness* to threat and pain perception.

### 4.1. Predisposing Risk Factor Trait Hypnotizaiblity and Blood Pressure Reactivity in the ICU from Cadiac Bypass Surgery

The rate of recovery and healing from the threat (HPAA) of cardiac bypass surgery was examined in 32 patients of *high*, *medium and low trait hypnotizability* in the ICU and hospital after all patients had received one session of hypnotherapy, delivered by a hypnotherapist *blinded* to the patients’ measured hypnotizability [22]. It was found that both *high (HH) and low (LH)*, but not moderate, hypnotizability was linked to *delayed recovery* and healing in the ICU and delayed hospital discharge from the threat (HPAA activation) by surgical incision (UCS-UCR) during coronary bypass surgery. High hypnotizable (HH) patients had more *labile blood pressure* in the ICU and required *more medication*, compared to the medium and low hypnotizable patients (*p* < 0.05). But high hypnotizable (HH) patients in the ICU through one session with a hypnotherapist appeared to *reduce or abolish* their perception of threat (HPAA) and *healed rapidly* in the ICU and were *discharged early* from the hospital. But *low hypnotizables* (LHs) who also had one session with the hypnotherapist (blinded to their trait hypnotizability) had *delayed recovery* from the surgery and were discharged later (mean = 5 days) from the hospital than the HH patients. *Moderate trait hypnotizability* patients, as predicted by the HRMTP, *stabilized more quickly* in the intensive care unit (ICU) than those who were *high or low* (*p* < 0.05) in trait hypnotizability. Measured trait hypnotizability was associated with the following *recovery sequence from surgery*: moderates, highs and lows. The above finding is consistent with a meta-analysis of 20 controlled studies of 1624 patients that found a large weighted effect size (D = 1.20; VarD = 0.83) and concluded that surgical patients in adjunctive hypnosis therapy had a better outcome than 89% of patients in control conditions [144]. The magnitude of this clinical efficacy finding in Montogomery et al. [144] was probably due to the confounding of the placebo effect (CS-CR) of hypnotherapy with the active ingredient trait hypnotizability (US-UR). The above [22] study supports the HRMTP precise prediction of quadratic modulation of human threat perception by trait hypnotizability.

### 4.2. Predisposing Risk Factor High and Low Hypnotizability Is Associated with Distinct Patterns of ANS Reactivity to Threat Induction

Jorgensen and Zachariae [23] in Denmark examined *ANS reactivity* to cognitive and emotional threat (HPAA) in *low*, *medium and high* trait hypnotizability in healthy college students. This research tested predictions derived from the HRMTP regarding high, medium and low trait hypnotizability and *ANS reactivity*. Hypnotizability was measured *9 months prior* to the ANS reactivity threat (HPAA) testing, and hypnotizability was never mentioned as relevant to the ANS threat (HPAA) testing conducted in a location and institution *different* from where the hypnotizability of the 71 healthy students (low H = 13, medium = 44 and high H = 14) was tested. This experimental context manipulation was performed to negate controversial hypothesized context effects [145] between the independent (trait hypnotizability) and dependent variables (ANS reactivity) in this study.

The investigators experimentally induced cognitive–emotional threat (HPAA) and measured the *sympathetic* and *parasympathetic* system (ANS) reactivity (EDA, ECG, HRV-HF-LF and BP) in 71 healthy college students, measured by a verbal report of stress, peripheral hand skin temperature, the electrodermal response—EDR—(mainly sympathetically innervated [84]) and high-frequency (HF) heart rate variability [146,147]. It is postulated that quadratically, trait a) high hypnotizability is associated with *Hyper*-responsiveness and B) trait low hypnotizability with *Hypo*-responsiveness of the neuroendocrine stress response axes (HPAA) and elevated or decreased sympathetic or parasympathetic basal tone. Electrodermal activity (*EDA)* was used as a measure of sympathetic activity and the high-frequency *(HF)* spectral component of heart rate variability as a measure of parasympathetic activity. High hypnotizables exhibited *greater EDA at baseline and slower EDA* recovery following both cognitive and emotional threat induction (HPAA) tasks than did medium and low hypnotizables (*p* = 0.003). “Medium hypnotizables responded with greater *decreases in normalized HF power* than did highs and lows during the emotional threat task. The results suggest diminished EDA variability in High hypnotizables and the potential for HF power as an indicator of *autonomic dysregulation in Low and High hypnotizables*, *compared to mediums*. In general, our results provide support for the hypothesis of differences in autonomic regulation between Low, Medium and High hypnotizable subjects, as predicted by the HRMTP. Our results could thus be interpreted as an indication of *autonomic dysregulation* in both Low and High hypnotizables, compared to *medium hypnotizables*, as predicted by the HRMTP” [23]. The above finding of potential ANS dysregulation in trait high and low hypnotizables is consistent with the prediction from the HRMTP [24,30,33,35,36,37,47,90].

### 4.3. Primary Predisposing Risk Factor Trait Hypnotizability–Suggestibility, Definition and Parameters

**Figure 2:**
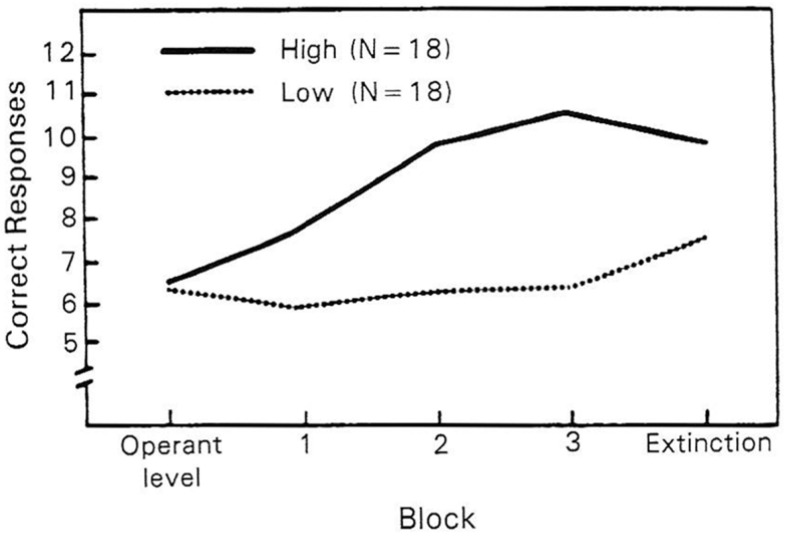
Verbal conditioning as a function of hypnotic susceptibility (Harvard). From “Hypnotic Susceptibility and Verbal Conditioning,” [27,156]. Reprinted from Wickramasekera, 1988 [27].

**Trait high hypnotizability** (HH) can be defined as the ***innate* ability** (US-UR) to modulate in **a dose–response or linear manner** human perception, cognition, memory, mood [65,110,148,149,150,151] and **ANS physiology** [152,153], with a **reduced** sense of **personal agency** or with the perception of “***involuntariness***” [154] in response to standardized **verbal suggestions** [65,110]. Trait high hypnotizability–suggestibility is also postulated to modulate ***automaticity*** in **adaptive or maladaptive human learning** [27,71,155,156,157,158,159,160] (Figure 2).

***Cognitive*** ***expectancy*** [110] appears to be an ***essential but not sufficient*** condition for **high hypnotizability as empirically shown by skeptics of trait hypnotizability Spanos et al., 1989** [161], **and Benham et al., 2006** [162]. Benham et al. [162,163] state, “There was an abundance of variance in hypnotic performance unexplained by the direct and indirect influence of **expectation** and compatible with the presence of an underlying cognitive ability” (p. 342). Horton & Crawford [164] apparently found that **increased** anterior corpus callosum size is positively correlated with trait **high hypnotizability** (HH). *Hypnotic induction* is associated with *decreased* activity in the *anterior default mode network* (DMN) ***only*** ***in trait high hypnotizable (HH)*** subjects [165] and naloxone does not block the **mechanism of hypnotic analgesia** in trait **HH** subjects [166]. **HHs** can modulate EEG-validated **REM and slow**-**wave sleep** in response to posthypnotic verbal suggestion [167,168].

Traits hypnotizability and suggestibility are **highly correlated** (r = 0.67–0.82) trait measures of personality with or without a hypnotic induction ritual [65,169,170]. Trait hypnotizability is measured with standardized *behavioral* tests like the Harvard Group scale or the Stanford scales, Forms A, B and *C **(Form C is the gold standard measure***). Trait hypnotizability is stable and has high (r = 0.71) **reliability over 15–25 years** [171] and appears in **behavioral data** to be **partly genetically based in monozygotic and dizygotic twins** [172,173], but in genetic data there is still controversy [174]. Trait hypnotizability is **orthogonal to the other Big Five trait factors in human personality** [175]. In the *general population* and also cross *cultures,* trait hypnotizability appears to be **normally distributed** [176]. But *trait hypnotizability* is apparently ***not*** ***normally distributed*** **in *psychopathology*** [30,34,38,51,177,178] but appears **quadratically distributed** in **somatization**, **functional somatic disorders or stress**-**related somatic symptoms**—SRSSs [22,27,30,32,33,37,38].

Trait hypnotizability–suggestibility, through a **verbal suggestion mechanism,** was found to robustly reduce both experimental and clinical pain perception in a **linear or dose–response manner**. Suggestions for the reduction in threatening clinical [40] and experimental pain [39] are modulated by the trait hypnotizability–suggestibility, as evidenced in **two large meta-analyses** of 42 controlled clinical pain studies and 85 controlled experimental pain studies. Trait hypnotizability–suggestibility is associated in clinical pain reduction with a mean weighted effect size of r = 0.53 (*p* < 0.001). Trait hypnotizability is associated in experimental pain with a 42% pain reduction in high hypnotizable (HH) and a 29% pain reduction in moderate hypnotizability and *negligible pain reduction in low hypnotizable (LH) people*.

The above finding is also consistent with a meta-analysis of 20 controlled studies of 1624 patients that found a large significant weighted effect size (D = 1.20) and concluded that surgical patients in adjunctive hypnosis therapy had a better outcome than 89% of patients in control conditions [144]. Rosendahl et al. [179] conducted a meta-analysis and concluded that effect sizes (medium to large) were largest for pain in threatening **invasive medical–surgical procedures** and **children/adolescents**. The magnitude of this clinical efficacy finding in the Montogomery et al. [144] study and the Rosendahl et al. study [179] was apparently due to the **confounding of the placebo effect (CS-US) of hypnotherapy** with the **unmeasured active ingredient trait (US-UR) hypnotizability–suggestibility** in the above two studies [33,53,54,61]. Innate trait **high and moderate** hypnotizability–suggestibility (US-UR) has a **reliable placebo effect** mobilized by verbal hypnotic induction **rituals** that should be **clinically leveraged**.

Recently, Landry et al. [180], and in a personal communication (2024), proposed that the most predictive *neural feature* identifying **high vs. low hypnotizability** in normal people was the aperiodic exponent of the EEG power spectrum measured at the anterior part of the frontal lobe at ***baseline* and outside of hypnosis verbal rituals** [181]. This suggests that trait hypnotizability may be a ***latent*** ***innate neural trait (US-UR)***, *prior* to any verbal hypnotic interventions. Previously, **baseline EEG theta** was found to discriminate between HH and LH normal people at baseline [71,182,183]. Significant gene–trait interactions have been studied in relation to attention and dopamine-related COMPT [174]. Cortade et al. [184] in a genetic study found that 89.5% of the individuals having the optimal COMT diplotypes had moderate to high hypnotizability (measurement with the Hypnotic Induction Profile, >3), based on a brief low-cost saliva test indicating a potential to identify a subset of primarily female patients (*p* < 0.001) (a relatively small number of men in this study) who may benefit from **medical–surgical** hypnosis interventions. Incidentally, the *COMT **amplifies stress reactivity through*** **cortisol** and the HPAA [185] and this is **consistent with prediction from the HRMTP**.

### 4.4. ANS Reactivity and Functional Somatic Disorders (FSDs)

**Figure 3:**
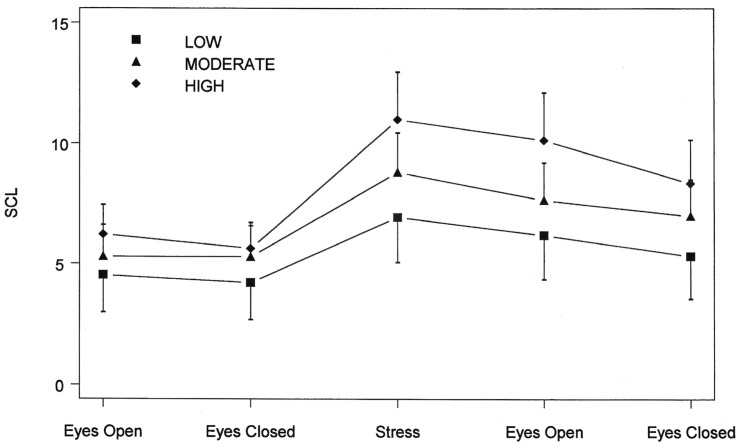
Hypnotic ability, SCL means and 95% confidence intervals (Wickramasekera et al., 1996 [36]).

A study (Figure 3) of 118 adult FSD patients presenting with **chronic pain and chronic somatic** symptoms found that **trait high hypnotizability** (Harvard test) modulated **experimentally** induced **threat** or negative affect, measured by their **electrodermal skin level** (SCL), in a **linear or dose–response manner** as hypothesized by the HRMTP. This hypothesized **interaction** of **hypnotic ability** and **negative affect** found that **larger increases** in the SCL during cognitive threat were **significantly related to higher levels of trait hypnotizability (*p* = 0.0065**). In addition, individuals with trait **high hypnotizability *retained* higher** levels of SCL (***delayed*** ***recovery*** from **threat** or stress) than individuals with **low hypnotic ability** (*p* = 0.0065) after cognitive stress. ANS-mediated **gastric acid** secretion [152] and ANS-mediated **pain** symptoms [153] have been shown to be **modulated** by trait hypnotizability–suggestibility. Also, trait hypnotizability apparently **modulates** muscular contraction or “**tension headache**” and vascular headache—**Migraine** [27,44,160], **Conversion Disorder** [45], moderate **obesity** [46], **morbid obesity** [47] and the **severity of somatic complaints** [34].

**High and low hypnotizability** is robustly (*p* < 0.001) and **quadratically** associated with FSD in patients (n = 83) with **chronic pain and multiple other somatic** symptoms in a medical school Behavioral Medicine Clinic and psychophysiology laboratory [30]. This study of chronic FSD patients was conducted on a consecutive series of 83 adult patients (male and female) presenting chronic pain and multiple chronic somatic symptoms (FSDs) ***without*** ***identified pathophysiology*** (investigated–tested previously **by multiple medical specialists and subspecialists)** who were **compared to a healthy matched** (mean age 40 years) community sample (N = 78) of male (N = 38) and female (N = 40) adults who were high, low and moderate in hypnotizability as defined by the Harvard Group test. The patient sample with **somatic symptoms** predicted by the HRMTP **was *quadratically*** distributed on **trait hypnotizability** and was significantly different from the *normal distribution* of high, low and moderate hypnotizability from the matched community control group (*p* < 0.001). **Trait low hypnotizability** was significantly (*p* < 0.003) more often associated with the presentation of **somatic** symptoms (pain, IBS, etc.) rather than **psychological** symptoms (anxiety, phobias and depression).

In two medical school sleep disorder clinics, **EEG**-**defined primary insomnia**, with *pathophysiology (apnea) and psychopathology* (depression) excluded, was **quadratically** and robustly (*p* < 0.0001) associated with trait **high and low hypnotizability** [38]. In high (HH) people, **EEG**-**defined slow**-**wave sleep (SWS)** was increased 81% by posthypnotic suggestion, and time spent awake was reduced by 67% [168]. In an early sleep laboratory study, **REM sleep was modulated** by posthypnotic suggestion ***only* i**n trait **high hypnotizable** normal subjects [167].

In a **meta-analysis** [41] of **functional neurological disorders**, trait high suggestibility was significantly (*p* < 0.001) associated with FND in a meta-analysis (FND: n = 316; control: n = 360) of FND patients. A systematic review [186] of 35 studies (N = 1584 patients) of the efficacy of hypnosis and suggestion for functional neurological disorders (FNDs) reported a **surprising 87% clinal efficacy rate** but did not measure or control for the patient-measured **innate trait hypnotizability–suggestibility,** which is the **implicated innate active ingredient (US-UR) in hypnotherapy** [33,53,54,61,71]. **Somatic complaint *severity*** in normal college students (n = 45) was positively correlated (r = 0.452; *p* < 0.002) with high hypnotizability (Waterloo–Stanford, Form C) as predicted by the HRMTP [34].

An unpublished PhD dissertation study [187] by an MD student of pulmonary function and HRMTP risk factors, in the hypnotherapy of **moderate to moderately severe asthma** patients, found that two risk factors of the HRMTP predicted the **magnitude of symptom and medication** reduction in these patients. Wagman [187] found that risk factors, 1. high hypnotizability (defined by *both* **Harvard, *p* < 0**.**0001, and Stanford Form C**, ***p* < 0**.**0001 measures**) and 2. High **Self**-**Deception** (*p* < 0.01) (Marlowe Crowne score >17) significantly (*p* < 0.001) predicted, with **pulmonary measurement**, the magnitude of ***medication*** ***reduction*** for these moderate to moderately severe asthma patients.

## 5. Can Automaticity and Reduced Sense of Self Agency in High Hypnotizable (HH) Patients Exponentially Amplify Threat Perception?

Amplified responsivity to suggestions in HH but not LH is based on evidence from the cognitive **gold standard Stroop test** [158,188,189,190] of ***automaticity*** in human information processing and cerebral EEG event-related potential-ERP data [157,191]. HHs appear to acquire and retain simple information very rapidly, and this *automaticity* in HH people’s information processing is not simply verbal but also extends down to the ***non-conscious*** ***perceptual level*** **as indicated by ERP data** ([157], personal communications) and is associated with greater ***cognitive*** ***flexibility*** in HH rather than in LH people in response to simple verbal instructions implicating the **anterior cingulate cortex** [110,159,190].

**Automaticity** is defined as processing information effortlessly, rapidly and ***involuntarily*** [192]. In a seminal experimental study, Raz et al. [158] showed that ***only*** ***in trait high hypnotizable (HH) people***, *with or without* a hypnotic induction, can a posthypnotic suggestion that ***words*** ***are meaningless or an alien language block normal automatic word recognition in*** proficient English readers using the **gold standard Stroop experimental procedure** [188,190]. The use of **verbal suggestion** to reliably ***alter*** ***the meaning of a noxious sensory stimulus*** (e.g., **surgical pain**) has been used for over 100 years in **clinical and experimental** hypnotic analgesia [27,63] and in numerous controlled meta-analytic studies with significant (D = 1.20) adjunctive hypnotherapy for **invasive** *medical diagnostic or surgical* procedures [144,179].

The HRMTP postulates that when **HHs** are **threatened** (HPAA) by **invasive physical or psychosocial** procedures (e.g., some clinical interviews), they may perceive cognitive-behavioral and motor alterations as occurring ***automatically*** ***and outside their voluntary control*** [157,190] and with a **reduced** sense of personal agency [154,193,194]. This perception in **trait high hypnotizables** of a reduced sense of agency [154] ***over*** ***one’s own mind and body can amplify*** **threat perception** (HPAA). This is particularly so in **HH** patients during ***interactions* with *other*** ***Predisposing risk factors***, like (1) **high verbal** **catastrophizing** and (2) **High trait Negative Affectivity** ruminations on past memories of grief–loss and adversity [27,90].

### 5.1. Trait Hypnotizability–Suggestibility and Related Trait Absorption (TAS) Modulate Learned Placebo and Nocebo Effects and Functional Somatic Disorders

**Figure 4:**
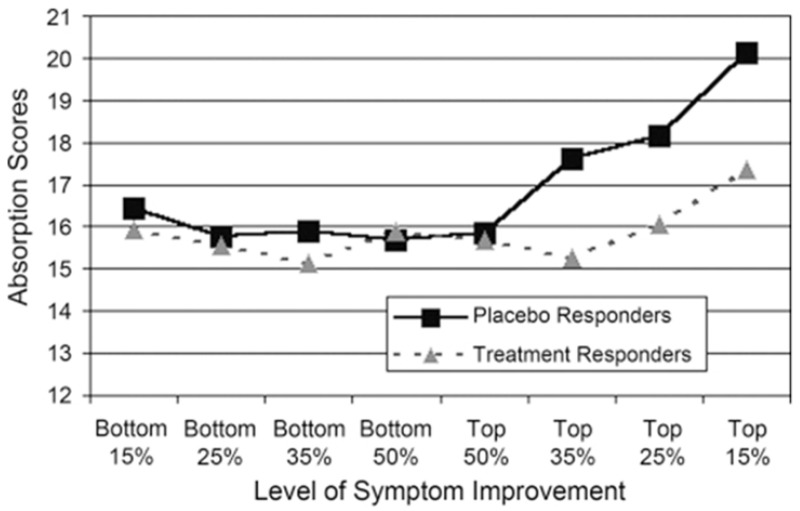
Absorption scores of placebo responders and treatment responders at increasing levels of symptom improvement (n = 16–56) [103].

Trait high hypnotizability–suggestibility and trait High Absorption are highly correlated, particularly in the high range of both trait Absorption and trait hypnotizability [195,196]. In a randomized double-blind placebo-controlled crossover study [103] of 117 Multiple Sclerosis patients, it was found that placebo responders scored significantly higher (*p* < 0.01) on trait Absorption than placebo non-responders, and discriminant analysis found that 80% of placebo responders were accurately identified (*p* < 0.0004) (Figure 4).

Trait hypnotizability–suggestibility is postulated [53,54,61,197] to modulate (1) **verbal suggestion learning** [39,40] and (2) **associative (CS-US) learning** of placebo effects [101,102,104,105] and **nocebo effects** [106,107,108,109].

Huber et al. [104] report an fMRI study of trait hypnotizability (*Stanford scale, Form A*) modulating brain activity linked to experimental placebo analgesia in brain-imaging data but ***not*** ***in behavioral*** **data**. In fact, they *did not use a formal hypnotic induction* procedure in this study because hypnotizability–suggestibility are highly (r = 0.67–0.87) correlated [170]. Also, this study acknowledged it was **deficient in high hypnotizable subjects** [104], and like Voudouris et al. [198], they **covertly reduced the intensity of the pain stimulus**. However, their study found that high hypnotizability (HH) was linked to increased analgesia after a *placebo conditioning procedure* and that this effect is mediated by ***decreased*** functional connectivity of the **DLPFC** with the **anterior cingulate**.

### 5.2. Trait Absorption (TAS) and Functional Somatic Disorders

My associates, I and others have used both the Harvard behavioral test of hypnotizability–suggestibility and *also* the psychometric *Tellegen Absorption scale (TAS)* [111] as a **surrogate measure** of hypnotizability–suggestibility in time-limited **medical–surgical** settings. Trait Absorption (TAS) can be defined as a person’s predisposition to become deeply attentionally engrossed in a ***sensory*** ***or imaginative experience*** [111]. TAS is a brief psychometric **noninvasive** test of hypnotizability–suggestibility, whereas the **invasive Harvard test** can be associated with an **invasive 5–10% incidence of somatic side effects**, particularly in **clinical samples** [27,199].

Trait Absorption (TAS) is a stable psychometric personality trait closely related to behavioral hypnotizability, despite the discrepant label trait Absorption [111,196,200]. The TAS measurement can be made in 15–20 min, unlike the Harvard and Stanford behavioral performance measures of trait hypnotizability that require specialized administration skills and require 1.5 to 2.5 h for testing and scoring. Controversially [145,201], personality traits hypnotizability–suggestibility and trait Absorption were claimed to be only **contextually correlated** [145], but spectral analysis of easy and challenging test suggestions of hypnotizability find that *high* hypnotizability and *High* Absorption are robustly correlated [196]. And in fact it was stated that “***Absorption*** ***may be a reasonably good predictor of the responses to the difficult cognitive suggestions***,” [196] and Balthazard & Woody [195] found Absorption related to the **hardest hypnotic tasks** (**hallucinations** and **cognitive distortions**) and a measure of **“true” hypnotic responsiveness**.

The TAS is stable and partly genetically based in ***behavioral*** ***data*** **in monozygotic and dizygotic twins *reared*** ***apart and togethe*****r** [202]. A heuristic and empirically supported distinction made by Tellegen [111]; personal communication, 1992) postulates that High Absorption (HA) people have an experiential or **respondent (Pavlovian)** mental set for human learning and Low Absorption (LA) people have an operant or **instrumental (Skinner)** mental set towards **learning**. This **theoretical distinction** appears supported by preliminary experimental and clinical studies in normal and clinical FSD samples [48,49,118,203,204,205].

TAS is significantly (*p* < 0.001) related to a wide range of phenomena, from morbid obesity surgical candidates [47] to **ANS**-**mediated anticipatory nausea and vomiting** both in and outside the **chemotherapy context** [42,43] and to risk factors for musical performance anxiety [112]. For example, High patient Absorption (TAS) is significantly (*p* ≤ 0.001) associated **with *anticipatory*** ***nausea and vomiting (CR) in chemotherapy patients*** [42] at the **sight or smell** of chemotherapy **nursing staff (CS**) both in and outside (e.g., **grocery store**) the clinical context of infusion and even in High TAS patients (N = 72) **remotely approaching** the clinical context (CS-US) of chemotherapy (US) infusion. This early correlational evidence of associative learning (CS-US) of neutral person-CS (e.g., nurses), place-CS (clinics and hospitals) and aversive **invasive** procedures (US-UR) associated with chemotherapy (CS-US) infusion ([53,54,206], NEJM) **automatically and unconsciously** eliciting anticipatory nausea and vomiting in High TAS patients has been replicated [43]. Trait Absorption (TAS) is associated (*p* < 0.01) with specific patterns of ANS reactivity (e.g., HRV and EDR) to cognitive experimental threat induction in normal college students [64]. Low TAS is robustly (*p* < 0.001) associated with morbid obesity in candidates for GI bypass surgery [47] and with **High and Low TAS** in functional somatic disorders (FSDs) in a primary care medical school clinic [27,50]. Also, it is associated with chronic pain in FSD patients in a private practice pain clinic [49] and is correlated with the magnitude of somatic symptom distress in a university Behavioral Medicine Clinic [48]. Vaitl et al. [207] reported a significant positive relationship between TAS and ***baroreflex sensitivity***. This modulation of cardiovascular baroreflex sensitivity by TAS has apparently been independently **replicated twice** [208]. It appears that the TAS measures at least a *cognitive–affective trait* that modulates **human *sensory* experiences** and may be related to **anomalous sensory experiences** across cultures and somatic symptoms [209]. High TAS appears to be associated with somatic symptoms in clinical and normal samples during threat (HPAA) perception, induced by **invasive physical and psychosocial procedures** [42,43,48,49,50,64]. In the thermal grill illusion test, amplified pain perception was associated with two HRMTP risk factors, Absorption and Neuroticism [203].

### 5.3. Predisposing Risk Factor Trait High Alexithymia Is Correlated Negatively with Low Hypnotizability and During Threat-Induced Functional Somatic Disorders

Trait **High Alexithymia** literally means “**without words for feelings**” [210] and is empirically linked to *emotional dysfunction* [81,143] and is also apparently linked to affective agnosia [78,80]. A seminal study by Frankel, Apfel-Savitz, Nemiah & Sifneos [72] reported an *inverse correlation* between two tests of trait hypnotizability (the Harvard test and the Hypnotic Induction Profile test of [211]) and an early measure of Alexithymia in a clinical (n = 32) sample, replicated three times with the same patients. This early inverse correlation between two measures of hypnotizability and Alexithymia was replicated by [74,75] with a larger normal sample (n = 286) and the present TAS-20 psychometric test of Alexithymia. Költő and Banyai [75] reported an inverse and more specifically a nonlinear or *quadratic* relationship between the Harvard and the TAS-20 scores.

Alexithymia is measured today with the Toronto Alexithymia scale (TAS-20), a psychometric scale which has high reliability and good construct validity [81,143]). **High Alexithymia** or High TAS-20 is associated with both **functional and organic somatic symptoms** [73] and with both a deficit in *emotional* processing and a surplus in *emotional* reactivity [73]. These claims about High Alexithymia and *emotionality* appear *problematic*. The TAS-20 uses verbal report measures of *beliefs* to measure three factors in Alexithymia: 1. deficits in identifying feelings, 2. deficits in describing feelings to other people and 3. a *constricted* imagination-fantasy process and *externally* oriented thinking (EOT). High Alexithymia appears empirically related *to **emotional dysregulation*** and insecure attachment to primary caregivers in childhood [81]. The above three personality features appear **congruent** with **low hypnotizability** [212].

A large mail survey using the TAS-20 and the Danish twin Registry (N = 8785) found that **genetic factors** have a similar impact on all three factors of Alexithymia [213]. Alexithymia (low hypnotizability) is strongly to moderately correlated with FSD or somatization [78,81,143,214,215,216]. The functional *somatic symptom and High Alexithymia link* appears to hold even in multiple brain-imaging studies [80,214].

**Affective agnosia** [78], a separate but related construct, is measured with the Levels of Emotional Awareness Scale—LEAS—and is a *performance*-based measure of a *deficit or absence of the ability to **experience feelings*** [78]. *The above two test measures of the emotional* domain correlate poorly [78]. Van Der Velde et al. [214], in a brain-imaging study, reported that High Alexithymia is associated with (1) stronger activation of the **anterior cingulate cortex (ACC**) during ***emotion*** processing, (2) *lower activation* in the *emotional attention* system and (3) reduced activation in areas of cognitive emotional processing. A meta-analysis of 258 patients found a significant association between Alexithymia and the risk of **delay in visits to the ER even during acute myocardial infarction** [217]. High Alexithymia is also reliably associated with a **lack of response to psychotherapy** [27,78,81]. FSD patients presenting IBS and High in Alexithymia (negatively correlated with low hypnotizability) respond to **invasive** rectal distention in a brain-imaging study with **strong *physiological*** ***reactivity*** in the brain’s **insula** but a **reduced subjective verbal report** of pain sensitivity [79,214]. The magnitude of this **incongruence *between*** ***verbal report and direct physiological measures*** **is predicted by the HRMTP to be larger during *high*** ***stressful*** **stimulation vs**. ***low*** ***stressful*** stimulation [26,27,69,70].

**Less** is known empirically about trait **low hypnotizability (LH) per se**, except for a propensity to **reduced cognitive flexibility** and **reduced *automatic*** ***or involuntary imagery*** as is apparently associated **with HH** [218]. According to the HRMTP, low hypnotizable people (on the Harvard and Stanford scales) are postulated to show an **incongruence between a) verbal report measures of threat and pain perception and b) direct physiological measures of pain and threat perception** (**EDR**, **frontalis EMG**, **BVP**, **HRV**, **BP**, **etc**.**)**. In low hypnotizable (LH) normal people, this ***postulated*** ***incongruence*** between verbal report measures vs. direct physiological measures (EMG, EDR and BP) *of **threat perception*** was empirically **first confirmed by Pomerantz & Wickramasekera (1992)** [70] and in Pomerantz’s (1986) [69] dissertation. As previously reported, traits hypnotizability and Alexithymia are **negatively correlated** in normal and clinical samples [75,177]. Many years later, **Gastroenterologists** Kano and Fukodo [216], using fMRI technology and the TAS-20 Alexithymia scale, appear to ***confirm* this early Pomerantz and Wickramasekera** [70] report with the blunt words, “The neural mechanism of Alexithymia is therefore activated more on a” ***physiologic***, ***motor-expressive level*** **and less in the cognitive-experiential domain of *the*** ***emotional response system***.” This ***incongruence*** between **objective physiological measures** and indirect verbal **subjective report measures** of pain during *high threat perception (rectal distention)* appear to link High Alexithymia [219] and low behaviorally (Harvard scale) measured hypnotizability [70]. It appears from the above review that **all three traits** (hypnotizability–suggestibility, Absorption and Alexithymia), despite ***discrepant*** ***labels***, **empirically converge** to predispose people to **FSD** as postulated by the HRMTP [27,30,33].

## 6. Is Pavlovian Learning of Biologically Embedded Threat Perception Modulated by the Predisposing, Triggering and Buffering Risk Factors of the HRMTP?

“*Stress is a state of mind, involving both the brain and body as well as their interactions; … it also reflects stable epigenetic modifications in development that set lifelong patterns of physiological reactivity and behavior through biological embedding of early environments interacting with cumulative change from experiences over the lifespan*.”

(McEwen, 2012, **PNAS**, p. 17180 [6])

Clinical threat learning [27,33,53,54,61] is postulated to be mediated by **unconscious automatic emotional** amygdala circuits [4,220] that ***precede*** ***laboratory-induced conscious cognitive expectancies and appraisal*** [221]. Jensen et al. [222,223,224] showed through associative learning and brain-imaging data that masked or ***unconscious*** neutral stimuli (CS) can ***automatically*** elicit placebo and nocebo responses and analgesic and hyperalgesic pain responses through ***unconscious*** ***neural mechanisms*** [224]. Hence, fMRI [222,223] and now behavioral data from Poland [225] show that placebo and nocebo effects can operate *automatically and unconsciously*, if *triggered* by the *context effects* of associative **neutral stimuli** (CS) of persons and places [33,53,54,61,226,227]. **Biologically embedded body memories** of unconscious learned threats (CS-US) in childhood [6,14,56,57] may be elicited by the HRMTP’s Triggering risk factors, adverse childhood experiences (ACEs) and/or stressful life experiences (SLEs) in adults [6,14,29,31,36,38,56,57,228].

These mechanisms of **biological embedding** include altered neuroendocrine stress regulation, chronic intermittent activation of the HPAA and apparent ANS dysregulation [35,37,94] and **stable altered gene expression** in methyl groups [14,228,229]. It is postulated based on ***strong*** ***correlative data*** but still incompletely understood **causal *epigenetic*** ***mechanisms (e***.***g***., heterochromatin and Euchromatin) that threat perception learning sets **lifelong patterns of physiological reactivity** and behavior through **biological embedding of the early childhood environment**, interacting with cumulative change through associative and other mechanisms of learning over the life span [6,7,29,37,56,228]. Biologically embedded risk factors like **ACEs and stressful life events (SLEs)** in adults appear to operate **automatically and unconsciously** if triggered by **present neutral stimuli** (CS) of nocebo experiences and are apparently based on stable changes in DNA methylation trajectories [6,56,228].

## 7. Are Predisposing Risk Factors Linked to Triggering Risk Factors of the HRMTP?

Brennan et al. [132] empirically showed that the **HRMTP**’s **Predisposing** risk factor (1) High trait Negative Affectivity links (*p* < 0.001) to **Triggering** risk factors (2) adverse childhood experiences (ACEs) and (3) adult **stressful life events**. These three risk factors of the HRMTP are postulated to be elicited by neutral CS ***unconsciously*** ***and automatically*** and linked through **associative learning** of threat perception [53,54,61,222,223,224,225] to present or **adult nocebo effects and FSD** [29,36]. In a groundbreaking nocebo effect study, **apparently implicating epigenetics**, Benedetti et al. [60] found that the rate and magnitude of nocebo somatic effects induced by **suggested threat** (HPAA) learning in 378 **heathy adults**, through an *inert or **“fake*** **placebo oxygen**” inhalation procedure, correlated with *maternal plasma cortisol* measured during the **first**, **second and third trimester** of maternal pregnancy. In these healthy adults, it was found that suggested high adverse event reports of somatic symptoms, in response to fake oxygen inhalation, including headache, chest pain, abdominal pain and objective cough, and **plasma cortisol responses** were positively correlated with intrauterine *maternal plasma cortisol*. If replicated, this *correlational* finding about maternal cortisol levels in the intrauterine milieu modulating nocebo hyperalgesia in high adverse event reports in adulthood [60] is consistent with the HRMTP’s hypothesis **of *unconscious*** ***and automatic*** **biologically embedded threat** [14] elicited by **neutral CS** (fake oxygen inhalation) Triggering biologically embedded risk factors like (1) adverse childhood events (ACEs) and (2) stressful life events in adults (SLEs) modulating present nocebo effects and FSD in adults.

### Respiratory Heart Rate Variability [19] Learning Activates and Modulates the Vagal Brake in Emotional Learning

It is known that relative to neutral (CS) sensory stimuli, attention and perceptual thresholds are biased towards **stimuli that convey threat** HPAA (UCS) to survival [230], and *attentional salience* appears critical in placebo and nocebo effects [231]. Placebo and nocebo somatic effects are **learned *hopeful*** ***or threatening*** **patient *emotional*** ***responses*** induced by **invasive** fake (inert, neutral or CS) medical procedures. Behavioral and neuroimaging data indicate both additive and interactive modulation of *trait and state emotional*-*affect* in the top-down central nervous system (CNS) modulation of human *attention* [232]. The HRMTP [30,31,33] postulates that *unconscious and automatically* Triggered *feelings* of (1) **safety**–**hope or** (2) **threat–fear** drive **clinical** placebo and nocebo *learning experiences* and FSD.

The HRMTP and Polyvagal theory [76,233,234,235] appears consistent with the ***primacy*** ***of human emotions*** **of *safety*** ***(placebo)*** **and *threat*** ***(nocebo)*** **in human learning** [30,53,61,236] in ***clinical*** situations, implicating **hormonal** changes [9] ***modulating*** ***human emotional behavior*** [135,236,237]. Polyvagal theory [76,233] proposes that unconscious neural evaluation (“neuroception”) of risk and safety reflexively triggers shifts in ***ANS*** ***states*** **without** requiring *conscious awareness **or cognitive appraisal*** [221,238]. Polyvagal theory also appears consistent with the **biological embedding** of threatening adverse childhood experiences [6,14]. Polyvagal theory [76,233] postulates that **social connectedness** and **trust** are a core biological imperative for human **survival**, since human survival particularly in the **health care domain** is dependent on trusted others (**parents and doctors**) and is wired into our genetics and is expressed through the life span from ***the*** ***moment of birth*** [233,234,235], mediated by the ***new*** ***ventral vagal*** **complex** [76,233]. There is apparently some controversy about the *neuroanatomical* basis of Polyvagal theory [239,240].

**Polyvagal theory** proposes that **trusting social engagements** are mediated through the **ventral vagal complex** and this complex has been empirically **linked to trait high hypnotizability** [241]. High-frequency (HF) heart rate variability (HRV) measures are related to **vagal modulation of heart rate** [146]. In a sample of normal nursing students, there was a strong relationship between measured (Harvard scale) trait hypnotizability and **both cardiac vagal tone (r = 0.45) and baseline heart rate (r = −0.47)** in the students [241]. These two variables were **strongly interrelated (r = −0.78)**. Thus, individuals with a lower heart rate and **greater cardiac vagal tone** were **higher in trait hypnotizability**. Blood pressure variables were not related to trait hypnotizability. Multiple regression analyses indicated that **approximately 40% of the individual difference variance** in **hypnotizability was accounted for by *baseline cardiac vagal tone*** and heart rate reactivity during **experimentally manipulated mood states** [151] in the students. **Heart-rate variability (HRV)** also appears to be **a quantitative measure of subjective self-reported hypnotic depth (SRHD) with normal people** [242] measured for trait hypnotizability with the brief clinical Hypnotic Induction Profile [243]. This small study (n = 10) found a significant **linear relationship** between SRHD and the **high frequency (HF) component of HRV**.

**High-frequency heart rate variability** (HF-HRV) **biofeedback training** delivered in a threatening context (a medical clinic) may temporarily reduce threat perception (HPAA) of the clinic through associative learning and **amplify safety perception** of the clinic and the patient’s emotional self-regulation [243,244,245,246]. HF-HRV biofeedback training [245] can be particularly effective with people of **low and moderate trait hypnotizability** (*approximately 85% of the general population*) **if delivered in conjunction with warmth and empathy** [61,71,247] to **increase their *objective* vagal tone, reduce SNS activation** and increase their **verbal–cognitive receptivity** to therapeutic verbal suggestion [33,61,71,151,241]. Oscillations in heart rate [248], an aspect of heart rate variability (HRV), show robust associations with **psychological health** [245,249,250] despite some **neuroanatomical** controversy related to Polyvagal theory [235,239,240,246].

Neural oscillations may interact directly with **slower** physiological rhythms, like heart rate and respiration [244]. Mather & Thayer [244] proposed that associations between HRV and ***psychological health*** may reflect a **causal** influence of cardiac rhythms on neural activity. Granger **causality** analyses indicate **stronger heart-to-brain than brain-to-heart effects** in **all frequency bands** except gamma [248] as originally proposed by Sir William Harvey in 1628 [27]. The above finding appears consistent with the ***reciprocal and bottom-up physiological regulation hypothesis*** [27,237] **and the *primacy of human emotions in placebo (safety and hope) and nocebo (threat–fear) clinical emotional learning*** [27,61,71,135,236,237].

## 8. Frequency of Functional Somatic Disorders in Primary Care Medicine and the Risk of Unintentional Iatrogenic Injury in the Invasive Medical–Surgical Hospital Context

“*Pain syndrome patients, in their desperate search for the elusive cure, often chase “windmills” and convince their doctors to perform a myriad of invasive tests, and procedures. As a result of their pain behaviors, many experience iatrogenic complications, suffering and disability*.”

—G.M. Aronoff, MD Editor, Clinical Journal of Pain, 1(1) 1985 [251].

Patients presenting with acute somatic nocebo effects [29] or chronic stress-related somatic symptoms (SRSSs) **without identified pathophysiology** [26,28,30,31,32] or FSD [55] are approximately **30–50%** of all presentations in **primary care medicine** [35,55,58,252,253]. Patients with chronic pain are known to *overutilize* medical diagnostic and surgical services to sometimes legitimize and be socially reinforced (empathy) and supported in their chronic disability and demands [27,59,138,253,254].

### Two Subsets of Patients at Risk for Unintentional Iatrogenic Injury

According to the HRMTP there appear to be at least ***two*** ***subsets*** of patients with chronic pain and somatic symptoms at risk of **overutilizing medical–surgical services**.

**The first subset** at risk, ***despite*** ***discrepant descriptive labels***, are trait **high hypnotizability–suggestibility** and related trait High Absorption patients, with ANS **sympathetic hyperactivity** to threat [21,22,23,34]. This first subset of FSD patients, **if low on Alexithymia and therapy**-**compliant**, is receptive and responsive to multiple forms of structured ***psychosocial*** therapy like CBT, EMDR, psychophysiological–biofeedback therapy [27,36,197] and pain reprocessing therapy [255] and have **moderate to high efficacy** rates in private and public pain clinics.

*The second* subset of FSD patients at risk for overutilizing medical–surgical services are postulated to be low on trait suggestibility–hypnotizability, Low on trait Absorption [111] and *High on trait Alexithymia*. According to the HRMTP, low hypnotizable people (on the Harvard and Stanford scales) are postulated to show emotional anomalies, such as an *incongruence* between (a) verbal report measures of threat and pain perception and (b) direct objective physiological measures of pain and threat perception (e.g., EDR, frontalis EMG, BVP, HRV, BP, etc.). The magnitude of this incongruence *between verbal report and direct physiological measures* is predicted by the HRMTP to be larger during *high stressful* stimulation vs. *low stressful* stimulation [26,27,69,70]. This second subset is associated with incongruities in ANS reactivity and perhaps affective agnosia [78,80], chronic somatic symptoms and ANS dysregulation [22,30,47,77,78,79,80,81,143,214,216]. This second subset is postulated by the HRMTP to be very likely insisting specifically on medical–surgical resolution of their pain or distressing somatic symptoms, even without pathophysiological findings [253,254,256]. These patients are postulated to be cognitively–affectively locked into a biomedical model [52] of their chronic pain or somatic symptoms. This low hypnotizability and High Alexithymia subset of FSD patients are cognitively–emotionally rigid and have a high dropout rate from conventional psychotherapy [27,73,81].

The diagnosis of FSD by inclusion of psychosocial risk factors (HRMTP) may reduce the risk of **unintentional iatrogenic injury** to patients with FSD, who are **insisting** on **medical–surgical resolution** of their chronic and distressing somatic symptoms [27,138,253,254]. Unintentional **iatrogenic injury** appears to be a leading cause of mortality in the USA [257,258,259,260]. A report in 2000 from the **Institute of Medicine**, **National Academy of Sciences** [257] found that 98,000 patients die in any given year from **unintentional medical errors** that occur in hospitals, which is more than died from motor vehicle accidents or breast cancer in 2000. Ten years later, Landrigan et al. in the ***NEJM*** [258], in a retrospective study of a stratified random sample of 10 hospitals in North Carolina from 2002 to 2007, found that multivariate analyses of ***hospital*** ***harms*** identified by internal reviewers showed ***no*** ***significant changes*** in the overall rate of harms per 1000 patient days from the earlier study [256]. Makary & Daniel [259], in a ***controversial*** ***paper*** in the *British Medical Journal*, reported that ***medical*** ***errors*** **were the *third*** ***leading cause of death*** in the USA. A recent apparent update on Medical Error Reduction and Prevention reports that medical errors are **still a leading cause of death** in the US [260]. It is likely that **some unknown percentage** of these patients **may be FSD**. In this busy context of technologically sophisticated medical care and scientific information compassionately applied, **learned** medical skills and science [7,261] battle the constraints of natural disease and death daily.

In the early years of **biofeedback therapy** [61,160], it was empirically established that there was little or **no correlation** between experimental alterations in **ANS or CNS physiological signals** (EMG, EEG, EDR and peripheral Blood Volume Pulse) and the efficacy of **clinical outcomes** [27,61,197,262,263,264]. It was clear that **non-specific factors** or **placebo effects** accounted for the **bulk of the variance** in the reduction in ***autonomically*** mediated clinical **somatic symptoms**. Hence, it was imperative to **specify the learning mechanisms of placebo somatic** effects [2,53,54,226] to **identify the psychosocial risk factors *modulating* placebo and nocebo somatic effects and somatization or FSD**. A promising and successful early effort to reduce **chronic pain** and **recruit non-specific suggestibility** effects was demonstrated by Ronald Melzack & Campbell Perry in an early salient paper in ***Experimental*** ***Neurology*** [265] and elsewhere [71,266,267].

## 9. The Conditioned Response Model (CRM) of Emotional Learning of Placebo and Nocebo Somatic Effects and FSD

It is postulated that **primitive** mechanisms of **threat**–**fear (nocebo)** and **safety–hope (placebo)** perception are associated with the **emotional learning**, implicated in the *context* of **invasively learned fake** placebo and nocebo **somatic effects** [10,12,53,54,61,197].

“*Wickramasekera was the first to propose a broad and coherent theoretical account of placebo effects as conditional reflexes*” [225]. Kirsch [221,268] challenged this view and proposed that **response expectancy** was a determinant of experience and behavior and **Placebo effects** and Colloca & Miller [269] accepted the expectancy model but expanded it to include conditioned stimuli and the **psychosocial context**. But **expectancies** are by definition ***conscious events*** and there is a large and growing body of empirical data establishing, under different labels (Classical conditioning, Nonconscious activation of placebo effects, Hidden conditioning, subliminal or low intensity sensory stimuli) providing evidence that **unconscious and automatic mechanisms** establish placebo and nocebo effects [222,224].

***Mechanisms***

1. Positive or negative **verbal suggestion** is postulated by the CRM to induce placebo or nocebo somatic effects and FSD, and **all invasive verbal suggestion** is postulated to be modulated by the **innately effective (US-UR) psychosocial active** ingredient **trait hypnotizability–suggestibility** [33,35,36,53,54,61,154,267,270]. Two large meta-analyses found that **trait hypnotizability–suggestibility modulates,** in a dose–response or *linear manner*, **the efficacy** of verbal suggestions to **reduce** *clinical and experimental* threat and pain perception [39,40].
2. Interpersonal delivery of rapport through accurate verbal Empathy and Warmth, are innately effective (US-UR) psychosocial active stimuli to induce and modulate placebo and nocebo somatic effects and FSD. Empirically, empathy and warmth can modulate anxiety, threat and pain perception [27,264,271,272,273,274,275,276]. It also appears that empathy is modulated by trait hypnotizability-suggestibility [247].
3. Pavlovian (CS-US) and other learning (operant, cognitive, observational, evaluative conditioning) is postulated to be modulated by trait Hypnotizability-Suggestibility [101,102,103,104,105,106,109,277].

**Predictions**:

1. The CRM predicts that the identification and application of **innately effective** [53,54,61,197] **psychosocially active stimuli** (US-UR), in the context of increasingly **invasive** [278] and **threatening** medical–surgical procedures (US-UR) on patients, can **amplify the magnitude of future placebo effects in clinical trials** [61,197,278,279,280,281,282,283].
2. **Associative learning** can, through **neutral stimuli** (CS), **automatically and unconsciously** [222,223] trigger activation or deactivation of **previously learned** (e. g., adverse childhood experiences and traumatic major life changes like the injury or death of a parent) and **biologically embedded nocebo effects** [6,7,28,29,31,35,37,54,56,228] through **epigenetic mechanisms,** like **heterochromatin and Euchromatin** [284,285,286].
3. **Trait *hypnotizability–suggestibility*** is postulated to be an **innately effective** (US-UR) modulator of **empathy learning** [247], **verbal suggestion learning** *and* **associative learning of *placebo*** ***somatic effects*** [101,102,103,104,105,106], **nocebo somatic** effects [106,107,108,109] and **functional somatic disorders** [23,30,33,34,41,44,45].
4. It is predicted by the CRM and HRMTP that **chronic anomalies** and **incongruities** [27,69,70,71] in **emotional perception and emotional learning and ANS reactivity** in trait **low hypnotizability–suggestibility and High Alexithymia** can **reduce HRV** [19] and increase risk of **ANS dysregulation** [28,30,31,35,36,79,80,81] as postulated in **chronic** threat and pain perception associated with allostasis and **biological embedding** [6,7,82].
5. It is predicted that trait **high hypnotizability (HH**) amplifies **threat** perception, and that HH is a risk factor for **FSD**, unless the patient’s HH **is mobilized specifically by verbal hypnotherapy or non-specifically by psychosocial therapies,** like pain reprocessing therapy, CBT, EMDR, or biofeedback therapy.

## 10. Clinical vs. Experimental Contexts

Recent studies suggest that **experimental** placebo effects may be **unstable across contexts (experimental vs. clinical)** but that **clinical** placebo effects as in **chronic pain patients** can be predicted by genetics, brain properties and language use [11]. Clinical associative **emotional learning** (CS-US) ***experiences*** are postulated by the Conditioned Response Model (CRM) of the placebo and nocebo somatic effects to be modulated by **trait high hypnotizability–suggestibility** [53,54,61], especially in ***interaction* with the Triggering risk factors** like adverse childhood experiences (ACEs) of the HRMTP. Placebo effects and pain perception can be significantly different in **clinical vs**. **experimental pain situations** because of the time- and place-**constrained** nature **of experimental pain** vs. the **ongoing aversive nature of clinical pain**. Nocebo threat learning frequently occurs in previous failed clinical ***experience* contexts** [226,227,287].

## 11. The Psychophysiology of the Clinician–Patient Relationship and Associative Emotional Learning Modulated by Trait Hypnotizability–Suggestibility and Social Support

The efficacy of the psychophysiology of healing, is postulated to be modulated by innately effective biological (US-UR) and psychosocial stimuli like empathy, warmth and the trait hypnotizability of persons as evidenced by behavioral and psychophysiological measures [9,264,272,273,274,276,288,289] and specifically linked to High Empathy and to trait High Hypnotizability [247]. Empathy and warmth were operationalized for measurement with psychometric scales [279] and now appear to be innately effective psycho-social stimuli (US-UR) for mental and somatic healing [27,276,290]. Social support [97,99,100,291] empathy and warmth are now increasingly supported by neural brain imaging data and operationalized by the specificity of perceived “interpersonal trust” [272,273,274,289]. An fMRI study found significant reduction in the neural systems supporting emotional and behavioral threat responses when women held their husband’s hand. Neural threat responses varied as a function of marital quality and were specific to the husband and but not a stranger [292]. Social support decreased pain-related skin conductance responses (EDR’s) in both women and men and social support reduced threat activation in neural data (fMRI) indicated by reduced nociceptive signals [274]. Doctor-patient warmth and empathy reduced nocebo effects and amplified placebo effects in a fake oxytocin nocebo and placebo study of 84 people [271]. In HH people, verbal suggestions reduce conflict in the brain and is associated with reduced anterior cingulate cortex activation (fMRI measure) but not in trait Low Hypnotizable (LH) people [189,293]. Theoretically, the Conditioned Response Model of associative emotional learning predicts that low empathy and low warmth [271] can negatively modulate the neutral stimuli (CS) of a doctor and an invasive medical procedure in a clinical context [33,53,54,61]. **Neutral stimuli (CS)** can elicit **unconscious and automatic** associative **threat** activation and nocebo effects [222,223,224] from any **Predisposing** (high catastrophizing) and **Triggering** (adverse childhood experiences) risk factors of the HRMTP in a specific patient.

Psychosocial active ingredients or innately effective stimuli (US-UR) like physician delivered (1) high empathy and (2) high warmth [271,276] in interaction with (3) High hypnotizability in the patient, can reduce or abolish a patient’s pain and threat perception [27,39,40]. These ***innately*** effective psychosocially **active** ingredients (US-UR) can be mobilized to **amplify the efficacy of verbal suggestion** to reduce nocebo somatic effects and to increase placebo effects [103,104,271] and to **reduce threat perception** that drives Functional Somatic Disorders [23,30,33,34,41,44,45].

International survey data of health care professionals (without measurement of trait hypnotizability) indicates that verbal hypnotic procedures [294] can amplify the efficacy of doctor-patient rapport induction procedures particularly in GI disorders [295].

## 12. General Predictions from the CRM and the HRMTP

1. The CRM predicted that **as new innate specific active biological ingredients** (US-UR), **including psychosocial active ingredients** (e.g., trait hypnotizability, empathy and warmth) for “healing” **are isolated**, paradoxically **the *placebo* respons*e* can get *stronger*** in future **randomized placebo-controlled gold standard clinical trials** [53,54,61]. This early prediction of **amplified placebo effects** in **future clinical trials** [53,54,61] appears to be supported today by the **high reported correlation** (r = 0.73) between **placebo effects and pharmacological effects** in recent clinical randomized controlled trials [281] predicted by Wickramasekera in 1980 and 1985. The increased magnitude of placebo effects in US clinical trials of **pain and especially of neuropathic pain** [282,296] support the **CRM prediction**. The progressive **mean increases** in placebo effects (40%) in Irritable Bowel Syndrome—IBS [297] are also supportive of the **CRM prediction**. The recent review of treatment effects in pharmacological randomized controlled clinical trials of **five diseases** [281] stated that the **placebo effect** is the “major driver of treatment effects in clinical trials that alone explains **69% of the variance**” [281]. This review [281] of the efficacy of 150 pharmacological randomized controlled clinical trials found that 72% of the variance in treatment effects could be attributed **to *placebo*** ***effects or context effects***.
2. The HRMTP predicts that, in FSD patients with chronic pain, intermittent threat perception can drive **dysregulation of the ANS and PTI**. This prediction was apparently confirmed by the association of experimental threat induction and Paradoxical Increase in hand Temperature (PTI) in approximately **50% of 224 of chronic pain patients of both genders**. This PTI was **first experimentally demonstrated by Wickramasekera et al.** [37] and **replicated independently** in a PhD dissertation [96].
3. The HRMTP predicts that the primary Predisposing risk factors (a) trait hypnotizability–suggestibility, if it implicates threat learning, ***interacting*** with other HRMTP risk factors (e.g., high catastrophizing, high ACEs and low social support) can **maladaptively** amplify threat perception in an **exponential** manner and launch a progressive trajectory of **severe morbidity, if not mortality**.
4. Acute nocebo effects may be the **maternity** ward of FSD [30,33,54]. Acute nocebo effects can become chronic FSD **resistant to specific medical–surgical therapy**.

## 13. Conclusions

Replicated clinical and even experimental studies in the last 50 years are supportive of the Conditioned Response Model’s [33,53,54] **three *mechanisms*** of **emotional learning** of clinical placebo and nocebo effects and FSD.

The High Risk Model of Threat Perception [24,30,33] predicts that the Predisposing, Triggering and Buffering psychosocial risk factors that modulate threat (HPAA) perception will also **modulate the *learning* of placebo**, **nocebo and functional somatic disorders (FSDs)**. Recent independent reviews [5,231,298] appear to confirm these predictions.

## Abbreviations

The following abbreviations are used in this manuscript:

HRMTP	High Risk Model of Threat Perception
CRM	Conditioned Response Model
FSD	Functional Somatic Disorder
HPAA	Hypothalamus–Pituitary–Adrenal Axis
ANS	Autonomic Nervous System
PTI	Paradoxical Hand Temperature Increase
SRSSs	Stress-Related Somatic Symptoms
US	Unconditioned Stimulus
UCR	Unconditioned Response
CS	Conditioned Stimulus
CR	Conditioned Response
